# Thermodynamic explanation of the universal correlation between oxygen evolution activity and corrosion of oxide catalysts

**DOI:** 10.1038/srep12167

**Published:** 2015-07-16

**Authors:** Tobias Binninger, Rhiyaad Mohamed, Kay Waltar, Emiliana Fabbri, Pieter Levecque, Rüdiger Kötz, Thomas J. Schmidt

**Affiliations:** 1 Paul Scherrer Institut, CH-5232 Villigen PSI, Switzerland; 2 HySA/Catalysis, University of Cape Town, Rondebosch, 7701, South Africa; 3 ETH Zürich, Laboratory of Physical Chemistry, CH-8093 Zürich, Switzerland

## Abstract

In recent years, the oxygen evolution reaction (OER) has attracted increased research interest due to its crucial role in electrochemical energy conversion devices for renewable energy applications. The vast majority of OER catalyst materials investigated are metal oxides of various compositions. The experimental results obtained on such materials strongly suggest the existence of a fundamental and universal correlation between the oxygen evolution activity and the corrosion of metal oxides. This corrosion manifests itself in structural changes and/or dissolution of the material. We prove from basic thermodynamic considerations that any metal oxide must become unstable under oxygen evolution conditions irrespective of the pH value. The reason is the thermodynamic instability of the oxygen anion in the metal oxide lattice. Our findings explain many of the experimentally observed corrosion phenomena on different metal oxide OER catalysts.

The oxygen evolution reaction (OER) on metal oxide catalysts has been a major focus in electrocatalysis research for several decades^1^,^2^,^3^,^4^,^5^,^6^. In recent years, interest in this field further increased due to the predicted key role of water electrolyzers in the development of an emission free hydrogen economy^7^,^8^,^9^. During the water electrolysis process, the oxygen evolving electrode performs with sluggish kinetics and is a limiting factor of electrolyzer voltage efficiency. In addition, most of the investigated metal oxide OER catalysts have been found to undergo substantial corrosion during OER, which limits the anode lifetime. Amongst the experimentally observed corrosion phenomena are the appearance of metal oxide lattice oxygen in the evolved oxygen gas^10^,^11^,^12^ dissolution of metal cations in the electrolyte during oxygen evolution^10^,^13^,^14^,^15^,^16^,^17^, and structural and compositional changes of the metal oxide catalyst with a loss of long-range crystalline order in a deeply reaching surface layer^15^,^18^,^19^,^20^,^21^,^22^. Most remarkably, in all cases a simultaneous onset of the investigated corrosion effect and oxygen evolution was observed. The substantial increase of the experimental evidence for metal oxide corrosion concomitant with oxygen evolution during the past few years has finally led to the hypothesis of a fundamental and universal correlation between metal oxide OER activity and metal oxide instability^14^,^15^. We will show in the following that this universal correlation can be understood from basic thermodynamic reasoning. The key role plays out in the oxygen evolution directly from the metal oxide crystal lattice, which we will term *lattice oxygen evolution reaction* (LOER) in the following.

## Results

The universal correlation between metal oxide instability and onset of oxygen evolution can be derived from thermodynamic principles. We will consider a general metal oxide of the type MO_n_, where n can also be a rational number, e.g. RuO_2_, IrO_2_, or Co_3_O_4_ ∫ CoO_4/3_. For practical reasons, we assume that the conductivity of the metal oxide is high enough to support charge transfer reactions. Furthermore, for simplicity, we will write all chemical equations in terms of the 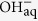 species for alkaline conditions. However, one should note that we can equivalently use the species 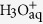 for acidic conditions by assuming that the chemical equilibrium of the water autoprotolysis holds. This also implies that the results derived in the following are independent of the pH value. An explicit reformulation of the equations for acidic conditions is presented in the Supplementary Information. For the following derivation, we only need to consider the three processes of

Chemical dissolution of the metal oxide without change of the cation oxidation state


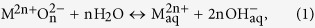


Oxygen evolution reaction (OER) from the electrolyte (water splitting)


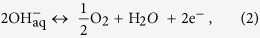


Lattice oxygen evolution reaction (LOER) directly from the metal oxide


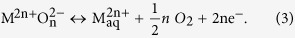


The restriction to these three processes does not preclude the possibility of other reactions like oxidation of the metal cation to a higher valency. Such additional processes do not alter the conclusions derived from the above and therefore do not have to be specifically considered.

The same reasoning can be applied to more complex metal oxide systems, e.g. perovskite oxides of composition ABO_3_ like SrRuO_3_. For such an oxide the chemical dissolution process could be written


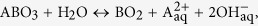


and the LOER


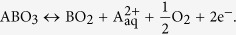


The chemical equilibria of the three processes (1), (2), and (3) can be written in terms of the different (electro)chemical potentials,

In these equations, we have exemplarily assumed that the A-site is occupied by an alkaline earth metal with valency 2+ and the B-site by a transition metal forming a stable oxide BO_2_. Of course, to be complete, we also have to take into account the chemical dissolution and the LOER of the oxide BO_2_ analogous to equations (1) and (3). However, this only adds to the complexity of the system of equations, but it does not change the conclusions derived thereof. Thus, the derivation of the instability of a perovskite under oxygen evolution conditions will be exactly analogous to the case of a metal oxide of the type MO_n_, which we will focus on in the following.


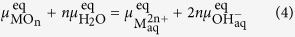



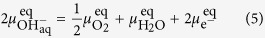



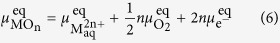


From these equations, it becomes obvious that at chemical equilibrium the three processes are not independent, but each of the three equations directly follows from the two others. Thus, at chemical equilibrium the lattice oxygen evolution reaction does not have to be considered independently because the respective equilibrium condition (6) is automatically fulfilled if equations (4) and (5) hold.

However, the situation drastically changes when we consider an applied overpotential in order to drive the oxygen evolution reaction (2). In this case, the OER is in a non-equilibrium state, and therefore equation (5) does not hold any more. The important consequence is that then also the other two equilibrium equations (4) and (6) cannot both be fulfilled at the same time any longer. This can be understood from a *reductio ad absurdum*: If these two equations would both still hold, then also equation (5) would mathematically follow in contradiction to the assumed non-equilibrium state of the OER. Thus, at least one of the two processes (1) and (3) will be also in a non-equilibrium state during OER.

When studying the OER, the concentration of the dissolved metal cation 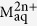 will be less than or at most equal to the equilibrium concentration defined by equation (4) for the following reason: At the moment of first contact between metal oxide electrode and electrolyte, the latter is free of metal cations. Subsequently, the metal cation concentration in the electrolyte can increase due to chemical dissolution of the metal oxide thereby approaching the equilibrium concentration from below. Therefore, the present (electro)chemical potential 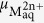 will always be smaller than or at most equal to the equilibrium chemical potential 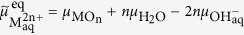 according to equation (4) with given (electro)chemical potentials 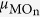, 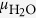, and 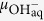. Thus,


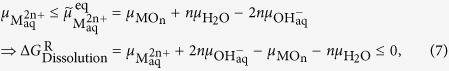


where we have introduced the partial molar Gibbs free energy of reaction 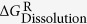 for the dissolution process. Please note that in this context we define the partial molar Gibbs free energy of reaction by the usual convention, Δ*G*^R^ = ∑ Δ*G*_products_ − ∑ Δ*G*_reactants_, meaning that the more negative Δ*G*^R^ the larger the thermodynamic driving force for this reaction. From equation (7), we can deduce that under oxygen evolution conditions, the thermodynamic driving force per mole of oxygen atoms 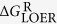 for the process of lattice oxygen evolution (3) is equal or even more negative than the thermodynamic driving force 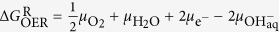 for oxygen evolution from the electrolyte (2):


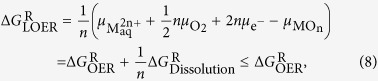


where we have used relation (7) and the fact that the (electro)chemical potential of a species is equal to the partial molar Gibbs free energy of that species. This proves the close thermodynamic correlation between OER and LOER. Relation (8) can be rewritten in terms of the respective overpotentials 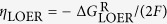 and 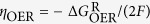 (where *F* is the Faraday constant):


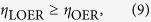


so that the LOER always feels a larger overpotential than the one applied for OER. As a remarkable consequence of this basic thermodynamic reasoning, a metal oxide cannot be stable under oxygen evolution conditions. This is not in contradiction to Poubaix diagrams of supposedly thermodynamically stable metal oxides like IrO_2_, because the process of oxygen evolution directly from the metal oxide lattice is not taken into account in these phase diagrams.

## Discussion

So far, we have derived that the bulk phase of any metal oxide is thermodynamically unstable at potentials above the oxygen evolution equilibrium potential. However, the experimentally observed corrosion phenomena were directly correlated with the actual *onset* of oxygen evolution on the respective metal oxide surface. Consequently, not only the thermodynamics of OER and LOER but also the kinetics of the two processes appear to be closely coupled. This could be understood from two points: Firstly, the distinction between adsorbed oxygen anions from the electrolyte on the surface of the metal oxide on the one hand and (ionically) bonded oxygen anions in lattice terminating surface rows on the other hand may become ambiguous. Secondly, the reaction pathways of LOER and OER could be expected to share common intermediate reaction steps towards O_2_ formation. This entanglement of the reaction mechanisms may explain the correlation between the actual onsets of OER and LOER on metal oxide surfaces.

**Figure 1:**
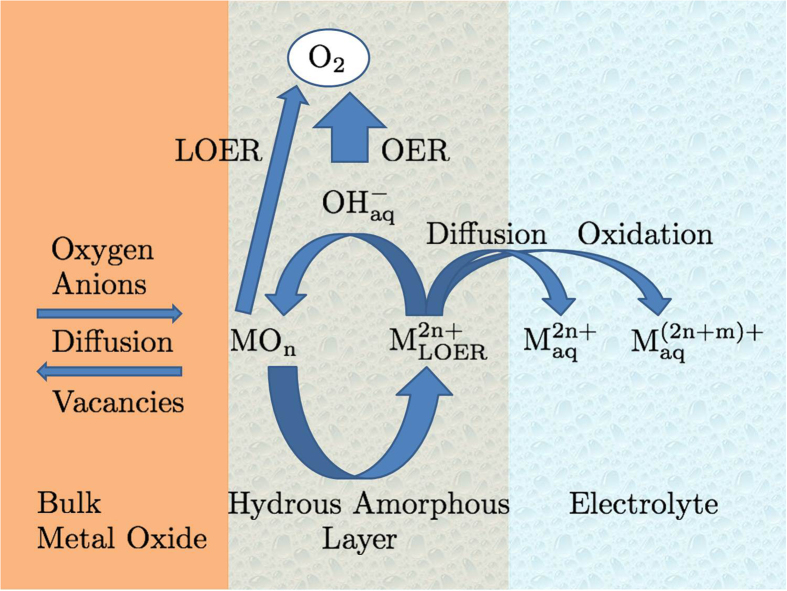
Schematic representation of the proposed LOER cation cycle. This cycle could lead to the formation of a 3-dimensional interface layer between the bulk metal oxide lattice and the electrolyte. The remaining metal cations from LOER can participate in the LOER cation cycle by recombination with aqueous oxygen anions or they can dissolve in the electrolyte either with unchanged valency or, after an additional oxidation step, in a higher valence state.

Figure 1 schematically depicts the different possible processes occurring as a consequence of LOER:

1. The remaining metal cation 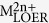 from LOER can dissolve in the electrolyte with unchanged valency and diffuse away from the electrode. This process will lead to a mass loss of the metal oxide electrode.

2. The metal cation 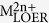 can be electrochemically oxidized to a higher valency state with higher solubility in the electrolyte,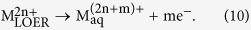 The resulting higher-valent cations can then dissolve in the electrolyte and diffuse away from the electrode. LOER followed by this process is equivalent to a simultaneous oxidation of the lattice oxygen anion and the lattice metal cation in one step,


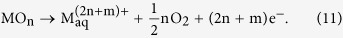


3. The metal cation 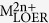 can recombine with hydroxide anions 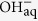 from the electrolyte to reform the metal oxide MO_n_ in analogy to the cation redeposition mechanism proposed in Ref. [23]. This process will close a cycle 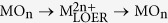 in the course of which n/2 oxygen molecules are evolved. This LOER cation cycle closely resembles the catalytic OER cycles proposed in the literature^24^,^25^,^26^,^27^,^28^,^29^. However, there are two important differences: First, the valency of the metal cation is *unchanged* during the LOER cation cycle, and second, the thermodynamic driving force for the LOER cation cycle is the oxidation of the lattice oxygen anion and not the oxidation of the cation to a higher valence state. It is likely that the “cloud” of metal cations participating in the LOER cation cycle forms a 3-dimensional boundary layer between bulk oxide and electrolyte with reduced structural order and rather undefined composition regarding the M:O stoichiometry. Such a boundary layer where the contrast between the solid metal oxide on the one side and the liquid electrolyte on the other side gets smeared out was proposed in the literature for hydrous metal oxide layers on anodized metal electrodes^30^. Vividly speaking, the surface of the metal oxide electrode starts to “boil” at the onset of OER and LOER. Furthermore, it can be hypothesized that water intrusion into that boundary layer is possible^18^. Therefore, it is termed “hydrous amorphous layer” in Fig. 1. In principle, the entire layer could contribute to the OER activity, thus forming a *3-dimensional active volume* similar to the situation in homogeneous catalysis^31^,^32^,^33^,^34^. This is in contrast to the usual perception of 2-dimensional active surfaces in electrocatalysis. In addition to the aforementioned parallels in the OER and LOER reaction mechanisms, this could be a further explanation for the higher OER activity of “less stable” metal oxides which are more prone to LOER and thus to the formation of such an active volume for OER. Due to the continuously driven LOER cation cycle, the hydrous amorphous layer will dynamically evolve during OER and can possibly grow into the bulk of the metal oxide. Although the LOER cation cycle could induce structural changes, it would not contribute to a mass loss of the metal oxide electrode. If the cycle was fully closed with all metal cations 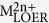 participating, the electrode could be considered to be in a dynamic but stable state. Only those cations leaving the cycle to get dissolved in the electrolyte and diffuse away from the electrode will generate an electrode mass loss.

4. The positively charged metal cations 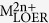 can extract oxygen anions from the bulk metal oxide lattice into the hydrous amorphous layer. This process is equivalent to a diffusion of thereby generated oxygen vacancies from the surface layer into the bulk and it can accelerate the 3-dimensional growth of the boundary layer.

Again, for perovskite oxides the situation is more complicated but analogous to the discussion above: For a perovskite ABO_3_, we have two remaining metal cations from LOER, A^p+^ and B^q+^. For many perovskites, the A-site cation is a soluble species and the B-site cation a rather insoluble transition metal species. Then 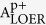 can follow the dissolution route whereas most of the 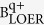 can participate in the LOER cation cycle leading to a hydrous amorphous layer enriched with B-species.

The results of the theoretical reasoning presented above agree very well with published experimental data about metal oxide OER catalyst activity and corrosion. Direct evolution of lattice oxygen was observed in several studies using oxygen isotope labelling in combination with on-line differential electrochemical mass spectrometry (DEMS)^10^,^11^,^12^. A direct correlation between OER and metal cation dissolution was found in several investigations by *in situ* differential reflectance spectroscopy, rotating ring-disc measurements, on-line DEMS, and inductively coupled plasma-mass spectroscopy^10^,^13^,^14^,^15^,^16^,^17^. Some of these studies discovered the dissolved metal cation in a higher oxidation state, which is in accordance with the subsequent oxidation of the metal cation after LOER, c.f. process (10). However, it should be noted that this correlation between the appearance of metal cations in a higher oxidation state and the onset of OER does not necessarily mean that these higher-valent cations are the active species for OER. Nevertheless, it also does not contradict this hypothesis. The proposed formation of a 3-dimensional hydrous amorphous layer due to the LOER cation cycle is in agreement with the experimental evidence of amorphization during OER observed on various oxides^15^,^18^,^19^,^20^,^21^,^22^. The fact that the amorphization of BSCF perovskite observed in Ref. [18] reached up to 20–100 nm deep into the bulk supports our theory of a thermodynamic instability of the bulk metal oxide phase as the cause of this amorphization effect.

Furthermore, the same LOER cation cycle could also occur during electrochemical oxide growth by anodization of metal electrodes at high potentials in the OER regime. At these conditions, the metal oxide phase is thermodynamically favoured over the metal phase, but at the same time LOER continuously drives the LOER cation cycle thereby preventing the growth of a highly-ordered crystalline bulk metal oxide. Vividly speaking, the metal oxide phase “wants to be but cannot be” at the same time. This might explain why such electrochemical oxides were observed to grow in a hydrous amorphous layer^14^,^31^,^35^ in contrast to the crystalline order of e.g. thermally grown metal oxides. The accessibility of more active sites within the 3-dimensional hydrous amorphous layer could explain the higher activity of amorphous electrochemical oxides compared with their crystalline form^14^,^22^ and the increase in the OER currents with consecutive potential cycles into the OER region as observed e.g. on certain perovskites^18^.

## Conclusion

We have derived the impossibility of a thermodynamically stable metal oxide under oxygen evolution conditions irrespective of the pH value. This universal instability of the bulk metal oxide phase explains many of the experimentally observed correlations between structural changes/dissolution of the metal oxide and the onset of OER. The basic driving force for the structural changes and the dissolution processes is the *instability of the oxygen anion* in the metal oxide lattice. Our findings are not in contradiction to Pourbaix diagrams, which predict the existence of many stable metal oxides at OER potentials. The reason is that common Pourbaix diagrams do not apply to oxygen evolution conditions because of the implicit assumption in a Pourbaix diagram that the oxygen partial pressure is in equilibrium at any potential. This is clearly not the case at OER conditions.

As a consequence, one possibility for technically stable OER metal oxide catalysts is the search for thermodynamically *meta-stable* metal oxides under OER conditions by decoupling the reaction rates of OER and LOER. One possibility to achieve such a decoupling would be the quest for metal oxides with an extremely low oxygen anion mobility in the bulk lattice. This will slow-down the diffusion of bulk oxygen anions to the surface and subsequently the LOER. Candidates for truly thermodynamically stable OER catalysts are *oxygen anion-free* salts containing anion species with a very high oxidation potential like fluorides, chlorides, or sulfates. Of course, the required electrical conductivity and the stability with respect to chemical dissolution are challenging criteria to be met by such materials.

## Additional Information

**How to cite this article**: Binninger, T. *et al*. Thermodynamic explanation of the universal correlation between oxygen evolution activity and corrosion of oxide catalysts. *Sci. Rep*. **5**, 12167; doi: 10.1038/srep12167 (2015).

## Supplementary Material

Supplementary Information
