# Supplementary material for: Thermodynamic explanation of the universal correlation between oxygen evolution activity and corrosion of oxide catalysts
Source: Sci Rep. 2015 Jul 16;5:12167. doi: 10.1038/srep12167 (PMC4503990; doi:10.1038/srep12167)
Supplement: Supplementary Information [file srep12167-s1.pdf]

Thermodynamic explanation of the universal correlation between oxygen evolution activity and corrosion of oxide catalysts

Tobias Binninger, Rhiyaad Mohamed, Kay Waltar, Emiliana Fabbri, Pieter Levecque, Rüdiger Kötz, and Thomas J. Schmidt

Supplementary Information

In the following, we will reformulate the three basic (electro)chemical processes (1)-(3) for acidic conditions with $H_3O^+_{aq}$ species.

Acidic conditions

1.) Chemical dissolution of the metal oxide without change of the cation oxidation state

$$\mathrm{M^{2n+}\,O_n^{2-} \quad + \quad 2n\,H_3O^+_{aq} \quad \longleftrightarrow \quad M^{2n+}_{aq} \quad + \quad 3n\,H_2O}\ ,$$

2.) Oxygen evolution reaction (OER) from the electrolyte (water splitting)

$$\mathrm{3\,H_2O \quad \longleftrightarrow \quad \frac{1}{2}\,O_2 \quad + \quad 2\,H_3O^+_{aq} \quad + \quad 2\,e^-}\ ,$$

3.) Lattice oxygen evolution reaction (LOER) directly from the metal oxide

$$\mathrm{M^{2n+}\,O_n^{2-} \quad \longleftrightarrow \quad M^{2n+}_{aq} \quad + \quad \frac{1}{2}n\,O_2 \quad + \quad 2n\,e^-}\ .$$

The consequent derivation of the thermodynamic instability of the metal oxide under OER conditions in acidic environment is exactly analogous to the one for alkaline environment.
